# Confidence and Hesitancy During the Early Roll-out of COVID-19 Vaccines Among Black, Hispanic, and Undocumented Immigrant Communities: a Review

**DOI:** 10.1007/s11524-021-00588-1

**Published:** 2021-12-23

**Authors:** SarahAnn M. McFadden, Jemal Demeke, Debbie Dada, Leo Wilton, Mengzu Wang, David Vlahov, LaRon E. Nelson

**Affiliations:** 1 Yale University School of Nursing, Orange, Campus Office #11501, P.O. Box 27399, West Haven, CT 06516-0972 USA; 2 St Michael’s Hospital Centre for Urban Health Solutions, Toronto, ON Canada; 3 Yale College, New Haven, CT USA; 4 State University of New York at Binghamton, Binghamton, NY USA; 5 School of Public Health, Yale University, New Haven, CT USA; 6 Science Applications International Corporation (“SAIC”), Reston, VA USA

## Abstract

Black and Hispanic Americans have been hardest hit with COVID-19 infections, hospitalizations, and deaths, yet during the first several months of vaccine roll-out they had the lowest level of vaccine uptake. Primarily, our research on vaccine hesitancy focused on skepticism around the vaccine itself and its roll-out. Our search strategy used PUBMED and Google with a prescribed set of definitions and search terms for two reasons: there were limited peer-reviewed studies during early period of roll-out and real-time perspectives were crucially needed. Literature searches occurred in April 2021and covered September 2020-April 2021. Analyses included expert opinion, survey results and qualitative summaries. Overall, for the general U.S. population, there was considerable hesitancy initially that remained high during the early roll-out. The general population expressed concerns over the speed of vaccine development (“warp speed”), confidence in the competence of government being involved in the development of vaccines and general mistrust of government. Among Black and Hispanic Americans, hesitancy was further expressed as mistrust in the medical establishment that was related to past and current medical mistreatment. Undocumented immigrants worried about access to insurance and possible deportation. These results on confidence in the vaccine early during vaccine roll-out suggest diverse reasons that influence a person’s decision to vaccinate or not. Additional barriers to vaccine uptake include complacency and access. To ensure health equity, particularly to address disparities in morbidity and mortality, vaccine hesitancy needs to be acknowledged and addressed as COVID-19 vaccine roll-out continues, and these observations calls for conscious planning to address these issues early with future health crises.

The Coronavirus Disease 2019 (COVID-19) pandemic has had a national impact in the USA. An early observation that has remained throughout the course of the COVID-19 pandemic has been the higher rates of test positivity[1], hospitalizations, and deaths among Black and Hispanic Americans and American Indian and Alaskan Natives compared to Whites and Asians [2–10]. While disparities have been documented across different stages of the pandemic, data updated through April 23, 2021 showed a decline in relative risk by racialized grouping and ethnicity with Whites as the reference racial group; this has been partly attributed to an increase in COVID-19 vaccination availability but a plateau in vaccine over time among Whites.

Reasons for the disparities in morbidity and mortality have been examined. Two early hypotheses were forwarded to explain higher rates of outcomes among Black, Hispanic, and American Indian/Native Alaskan persons. Firstly, population density with a greater likelihood of crowding into housing could result in greater viral transmission, and the second was that a higher prevalence of underlying chronic medical conditions could account for increased risk of hospitalizations and death [11]. Moreover, an important source of transmission that initially received less attention was the higher proportion of racialized minorities engaged in lower paying “essential services” and critical infrastructure positions in exposed industries. These positions included health care support and such public facing front line positions as in food preparation and serving, grocery stores, building and ground cleaning, and transportation and material moving [12]. Distribution and uptake of personal protective equipment came late to these settings. Due to economic consequences of social distancing and local public impatience with adherence to behavioral public health interventions, essential workers could become exposed to infected persons through close contact. These interactions were sometimes exacerbated by violence from the public that resented admonitions to comply with mask orders.

A major advance in the control of the COVID-19 pandemic has been the development and rollout of highly effective vaccines [13–15]. Despite the sustained excess of cases, hospitalizations and deaths among Black, Hispanic, and Native American/Indigenous persons, rates of COVID-19 vaccine uptake have lagged behind that of Whites [16]. While uptake increased among all groups in March and April 2021, vaccine uptake continued to be lower among Black and Hispanic persons compared to Whites and Asians. This report also showed that the disparity increased over time. While Black and Hispanic persons have been more likely to be counted as cases, hospitalized, deaths, and rates of vaccine uptake remained the lowest.

The purpose of this review is to examine the literature on vaccine hesitancy, acceptance, and intention. Given the profound disparities of cases, hospitalizations, and deaths by these social, cultural, and civic groupings, these factors are our primary focus. The first step was to summarize disparities in vaccine acceptance and uptake and then to identify key factors that shape those differences. While the issues of vaccine hesitancy and uptake are not new to the recent release of COVID-19 vaccine, the circumstances of this rapidly unfolding widespread public health crisis are different from previous bioevents. Lessons learned can provide insights into planning for future bio-events.

## Methods

Due to the urgency of this topic, we developed a search methodology that could capture the state of science, including published peer-reviewed research and reports on vaccine hesitancy, acceptance, intent, and uptake. Literature searches occurred in April 2021 and covered September 2020 to April 2021. We extended our search to include the mainstream media, commentaries, organizational reports using primary data or secondary analyses, and interviews.

Search engines for this review were PubMed and Google Search. Primary search terms used on both platforms include: (“COVID-19 vaccine hesitancy” OR “COVID-19 vaccine acceptance” OR “COVID-19 intention” OR “COVID-19 misinformation” OR “COVID-19 vaccine trust”) AND (race OR ethnicity OR immigration status) AND USA. To ensure that this search was comprehensive, we used snowball methods for secondary search terms [17]. These included “Black,” “Hispanic,” “Latino,” “Asian,” “Native American,” “Indigenous,” “Pacific Islander.” Note that in reporting, most articles used the term Hispanic so this term rather than Latino/Latina/Latinx was used in the narrative. In Google Search, only the first five pages of results (i.e., 50 links) sorted by relevance were included.

### Inclusion/Exclusion Criteria

Publications were limited to the USA, written in English, and published during and after September 2020. The rationale for the time frame was that reports before this time examined population knowledge, attitudes, and intentions for a hypothetical COVID-19 vaccine, whereas literature available since September 2020 were more likely to have the context of imminent availability and distribution of a COVID-19 vaccine. Abstracts only were excluded. Media stories or commentaries that were only about content from an otherwise published report without any additional analyses were excluded in favor of the original referenced material.

### Data Abstraction

Each primary search term was assigned to an individual team member with a second member replicating the search. Data abstraction included sourcing of data: primary or secondary. Line listings captured the following characteristics: author, title, journal or website (with date accessed), date of publication, literature type (e.g., journal article, newspaper article). In addition, to address all the outcomes in this review, we created columns with indicators (“1” or “0”) for availability of information on equity, interventions, communication strategies, logistics, vaccine hesitancy, vaccine uptake, respectively. Categories were created to denote the specific group(s) discussed in the article based on race, ethnicity, and immigration status. Study methods were recorded: sample strategy and final size, study design, data collection, and analytic methods. Key findings were recorded. Validation (independently by DV and SAM) for both PubMed and Google searches were performed for select search terms to examine for relevance, significance, and validity for study inclusion.

### Analyses and Reporting

Study findings in accordance with the outcomes were reported by (authors) DD, JD, LT, SAM, and DV using narratives and tables. Data were grouped by their outcomes and study populations (i.e., demographic search terms). We include a disclaimer that we are using categories from the survey tools. We conducted a narrative synthesis of the data to identify common findings (Table 1).

## Results

For all searches, 298 records were identified through PubMed, and a further 356 were identified through a Google search (Table 3). Of the 658 total articles identified, 215 were included, and 443 were excluded because they were either duplicates or did not meet the inclusion criteria. Table 2 shows search details for each demographic group.

**Table 1:** Literature search on COVID-19 vaccine hesitancy by demographic group, September 2020 to April, 2021

	Initial search						Snowball	
	PubMed			Google			PubMed	Google
	Total	Included	Excluded	Total	Included	Excluded		
Ethnicity	85	37	48	153	65	88	0	0
Race	213	41	172	153	47	106	0	0
Immigration	0	0	0	50	21	29	1	3
**Total**	**298**	**78**	**220**	**356**	**133**	**223**	**1**	**3**

**Table 2:** Rationale expressed for COVID-19 vaccine hesitancy by population group, June 2020 to April 2021

Population group	Rationale
Overall population	• Lack of trust in government • Vaccines developed too quickly • Vaccine safety • Wait and see • Fear of injection • Lack time and money • Low risk, so do not need it
Black	• History of medical abuse • Government cannot be trusted • Experience of racism in medical settings – treated different • Probably cannot get vaccine from a place they trust • Time off from work to get the vaccine • Lost time from work because of side effects • Catch COVID-19 from the vaccine
Hispanic	• History of medical abuse • Implicit bias against Hispanics • Not sick, perceive self to be healthy • Insufficient information in Spanish • Concern about possible deportation from data collected
Immigrants	• Concern about possible deportation if vaccine is defined as a public charge • Cost of vaccine • Side effects may affect ability to work • DNA alteration that forces sterilization • Language barrier

### Vaccine Hesitancy U.S. Population Overall

We first considered vaccine hesitancy for the overall U.S. population to describe general trends. We examined multiple national surveys from established survey organizations that reported a calendar sequence using consistent methods over time. A sequence of NPR/Marist polls through the Spring of 2021 showed that one-third of the sample representing the overall U.S. population responded that they would not choose to be vaccinated or were unsure (Fig. 2). While the proportion of those not choosing or who were unsure about taking the vaccine declined modestly after the vaccine became available; changes since that time were minimal [18]. The Gallup polls over the same period show somewhat fewer who would decline the vaccine (January 2021: 29%; March 21, 2021: 26% with March data being 2 weeks after NPR/Marist March poll), results on trends were similar in Gallup polls conducted over the same periods [19].

The Kaiser Family Foundation framed different response categories (“already got,” “as soon as possible,” “wait and see,” “only if required,” “definitely not”), but the trend of those reporting “definitely not” remained similar over the same calendar time frame [20]. Pew Charitable Trust reported a similar pattern over time [21].

Reasons for hesitancy or lack of intent to get the vaccine were wide ranging. In a December 2020 national survey, 50.9% indicated that they did not intend to be vaccinated. Younger adults, women, non-Hispanic Black (Black) persons, adults living in nonmetropolitan areas, and adults with lower educational attainment, with lower income, and without health insurance were most likely to report lack of intent to receive COVID-19 vaccine. The most frequently cited reasons were concerns about side effects and safety of the COVID-19 vaccine (29.8%), planning to wait to see if the vaccine is safe and consider receiving it later (14.5%), lack of trust in the government (12.5%), and concern that COVID-19 vaccines were developed too quickly (10.4%) [22] Table 1.

We examined views in the early months of vaccine roll-out from health care workers who are trusted sources of information and persuasion regarding the vaccine [23]. Although we found no national survey an October/November 2020 online survey of health care workers in five large midwestern health care systems reported that 56% were unsure or would wait to review more data and 8% did not plan to get the vaccine. Safety (69%), effectiveness (69%), and speed of development/ approval (74%) were noted as the most common concerns regarding COVID-19 vaccination [24]. In November/December 2020 in Philadelphia as vaccine was becoming available, 63.7% of employees reported that they planned to receive a COVID-19 vaccine, 26.3% were unsure, and 10.0% did not plan to be vaccinated. Over 80% of those unsure or unwilling to be vaccinated expressed concerns about vaccine side effects and the vaccines' newness.

### Vaccine Hesitancy by Demographic Groups

Given this report’s focus on information on factors that influence vaccine related disparities in morbidity and mortality, examination of population-specific groups follows. Our focus on understanding patterns by race and ethnicity is not an endorsement for the notion of clinically meaningful biological difference between these groups, but our recognition that in the USA skin color and shared cultural practices have been used as bases to construct categories to which people become de facto assigned. These social categories of difference have social and material consequences precisely because they are used to distribute both privilege and disadvantage, in ways that mediate exposure to (or protection from) health risks—including to COVID-19.

### Race

#### Black Americans

In most studies, Black respondents had higher rates of COVID-19 vaccine hesitancy than White respondents, but these differences declined over time [18]. In a June 2020 national survey, Khubchandani et al. [25] noted that vaccine hesitancy was higher in Black Americans than White and Asian Americans (34%, 22%, and 11%, respectively). Notably, this survey provided data on Asian Americans; a group not frequently presented in other reports. In November 2020, a Pew Foundation study reported that Black respondents were more likely than White, Hispanic, or Asian respondents to say “definitely no” or “probably no” to getting a COVID-19 vaccine (58%, 39%, 37%, and 17%, respectively). In December 2020, a national survey demonstrated that Black adults were more likely than White and Hispanic adults to respond that they would “probably not” or “definitely not” get vaccinated (49%, 34%, 32%, respectively) [26]. In January–February 2021, another nationally representative survey reported Black with non-Hispanic identity, low education and low income were each independently associated with a lower likelihood of definitively planning to get vaccinated [27]. A survey of health care workers (HCWs) at two large academic hospitals found vaccine hesitancy highest among Black HCWs (83%) compared to White HCWs (46.2) and Asian HCWs (47.1%) [28]. More recent data from an NPR/Marist poll in March 2021 show that differences in vaccine hesitancy by race had started to converge (Black: 25%; White: 28%) but remained elevated for Hispanic ethnicity (37%) [18]. However, despite reduced hesitancy, Black Americans were not being vaccinated at the same rate as Whites. Black non-Hispanics were 52% less likely than White non-Hispanics to get the COVID vaccine, according to surveys conducted from January to March 2021 [27].

As the disparities of COVID-19 vaccine hesitancy between Black and White Americans have started to narrow (due to increase in acceptance among Black people and a leveling or decline in White peoples’ acceptance from May 2020 to March 2021) [21], overall one-third remained resistant to vaccine acceptance in March 2021 [21]. Similar results were reported from an ABC/Washington Post poll in late April 2021 where between January and April, the positive difference in those who are vaccinated or those inclined to be vaccinated rose more for Hispanics and Black Americans than Whites (16%, 11%, and 5%, respectively) [29, 30].

#### Reasons for Hesitancy Among Black Americans

Data from the early rollout period of the COVID-19 vaccine document differences in reasoning for hesitancy by race. Black participants displayed lower trust than Whites in relation to government and the medical professionals; however, their hesitancy was more about the government’s motives rather than its competence [31].

Vaccine hesitancy in the Black community is a well-documented phenomenon in public health literature, but if the narrative starts there, the lesson of the situation is obscured. Much distrust has been attributed to the legacy of egregious and unethical events in American public health history, such as the unethical US Public Health Service syphilis study conducted in Black men in Tuskegee Alabama [32]. However, as medical historian Susan Everby stated in reference to the COVID-19 pandemic, “it’s not just the history, it’s the lived reality of everyday life that people experience in racism that makes the hesitancy come through” [33]. A 2016 study showed that many white medical students still wrongly believed that African Americans have a higher tolerance for pain [34]. Environmental barriers to all vaccine access include medical deserts in predominantly Black communities, transportation access to health clinics, and health insurance access. It is therefore experiences of structural and overt racism that influence Black Americans’ increased likelihood of distrusting the medical establishment and being COVID-19 vaccine hesitant. As noted in an editorial from the *Journal of National Medical Association*, the official premiere journal of the organization representing Black American physicians, referring to earlier experience with influenza vaccine program, “African Americans continue to experience low vaccination uptake, stemming, at least in part, from years of bias in and mistrust of orthodox medicine, safety concerns, and environmental barriers to vaccine access” [35].

In a RAND report on Black Americans from the American Life Study survey conducted in late 2020, 30% of Black respondents reported being vaccine hesitant [36]. As stated in their report, mistrust in the government around COVID-19, as well as perceptions of racism and unequal treatment in health care around COVID-19, were high. To summarize, 64% of all respondents agreed or strongly agreed that “A lot of information about COVID-19 is being held back by the government,” 59% of all respondents agreed or strongly agreed that “The government cannot be trusted to tell the truth about COVID-19,” and 59% of all respondents agreed or strongly agreed that “People who take a COVID-19 vaccine will be like human guinea pigs.” With respect to health care workers, 64% did not agree that “When it comes to COVID-19, Black people will receive the same medical care from health care providers as people from other groups,” 63% agreed or strongly agreed that “Within the health care system, people from my racial/ethnic group are treated differently than people from other groups.” In a separate report, another reason offered for the disparity in vaccine hesitancy was not having inclusive participation for COVID-19 clinical research. Inclusive participation can “address misinformation and distrust around the FDA-authorized vaccines to promote uptake” [37].

In April 2021, Kaiser Family Foundation released an update of their COVID-19 vaccine monitor [38]. Findings are similar with vaccine hesitancy influenced by mistrust of the health care system, concern for side effects, and time away from work related to the vaccine appointment as well as potential side effects [38]. To address these concerns, many of which were expressed more frequently among racialized minority groups, respondents wanted better access to information as well as the ability to obtain the vaccine at trusted places.

Notably, a survey of Black health care workers in late 2020 found that hesitancy was not attributed to history of racist practices. “Concern about side effects,” “vaccine is too new,” and “don’t know enough about the vaccine” ranked the highest as reasons for vaccine hesitancy for Black health care workers, which was the same for White, Asian, and Hispanic HCWs [24].

#### Asian American/Pacific Islanders

Asian Americans had rates of COVID-19 cases and deaths that were lower than White Americans, and rates of vaccine uptake have been similar or higher than White Americans [16, 39]. Reports on vaccine hesitancy make two points. In one national survey of Asian Americans/Pacific Islanders (composition 97.6% and 2.4%, respectively) conducted October–December, 2020, questions were posed about vaccine side effects, safety, religious reasons, belief in natural remedies, against vaccines in general, and fear of needles. Overall, 76% of 1646 had at least one concern about the vaccine with two-thirds expressing concern about side effects and 2% around safety; other reasons were lumped under “other.” Notably, on 2.5% would definitely reject a COVID-19 vaccine if offered. Level of concern differed between Vietnamese and Chinese Americans suggesting diversity of views among East Asian ethnicities [40]. A report from Texas raised the issue of language barriers and access to technology as barriers to uptake that need to be addressed [41].

#### Indigenous Peoples

With the highest risk of hospitalizations and second for deaths, indigenous peoples of the North American continent (American Indian/ Alaska Native) showed high levels of vaccine acceptance. In one online study of 1435 people from 318 tribal affiliations, 75% said they would take a COVID-19 vaccine; of those who were hesitant, the main concern was about side effects (89% of the unwilling) [42]. The reason most often stated for taking the vaccine was to do it for the community, but barriers to vaccination, such as transportation, were also noted [42]. Data on Pacific Islanders/Native Hawaiians are sparse and were included only in surveys of Asian Americans.

### Ethnicity

Hispanic Americans have consistently experienced excess risk compared to White Americans on COVID-19 cases, hospitalizations, and deaths [3]. Yet, rates of vaccine uptake lagged behind Whites between January and March 2021, with differences expanding during this early period of observation [38].

Although risk of COVID-19 outcomes was worse for Black and Hispanic than White Americans, attention on vaccine hesitancy has focused more on Black communities. Studies on COVID-19 vaccine hesitancy involving Hispanics also lagged behind that of Black Americans. In the 6 months between September 2020 and March 2021, PUBMED had 10 articles which included Hispanic Americans versus 50 that addressed Black Americans. In the 10 articles that mentioned Hispanic Americans, none focused solely on Hispanic people; rather, they were reported as stratum in a broader paper on race and ethnicity. No papers on PUBMED were exclusively devoted to Hispanics during the search phase. Searches on Google were predominantly news reports on local efforts. Some reports did not mention Hispanics in the abstracts and some not even in the narrative (e.g., CDC) [22].

When reported, rates of vaccine hesitancy before September 2020 have been higher among Hispanic Americans than White Americans [25, 43–45]. In September 2020, an exception was among Hispanic Americans in North Carolina who reported lower levels of hesitancy than Black and White Americans [45]. By November/December 2020, hesitancy (combined response categories of “definitely not” and “probably not get it”) was 35% for Black, 26% for Hispanic, and 26% for White people. Among Hispanic Americans, sub-analyses showed rates were higher for those 18–49 year old than those 50 and older, while essential workers expressed more reservations [46]. In March 2021, the “definitely no” response for Hispanic Americans was more similar to White than Black Americans (i.e., odds ratio with Whites as the reference group: Hispanic 1.42, Black 3.15) [47]. As reports on levels of hesitancy were sparse, similarly too was information of reasons driving hesitancy in Hispanics.

Hispanic Americans also have experience medical distrust based on ongoing and historical medical mistreatment. In one study from the 1940s, sex workers were used to expose prisoners in Guatemalan jails with sexually transmitted infections [48]. In the 1950s, Puerto Rican women from low-income communities were given experimental birth control pills without being told they were part of a clinical trial [49]. Sterilization without consent was practiced in Los Angeles until the end of the 1970s [50].

Discussions current for this review’s window had limited focus on past injustices and instead prioritize the urgent issues of access and approaches to service delivery. In one study [51], levels of hesitancy were similar to findings from studies of Black and White communities; however, the reasons given for not getting vaccinated were lack of time or money, fear of injections and side effects, and lack of interest or motivation. Others did not get vaccines because they perceived themselves to be healthy and did not feel sick. A related issue is insufficient information where there has been limited access to COVID-19 vaccine information that is in Spanish [52].

A salient politico-legal issue was about documented status and concern about possible deportation. The concern was about attending a clinic and having their personally identifiable information entered into a database and possibly having those data shared with the federal law enforcement agencies. Another concern was the lack of health insurance among Hispanics, even though nationally, anyone could receive the COVID-19 vaccine regardless of insurance coverage or immigration status.

### Undocumented Immigrants

Few reports quantified the effect of immigration status (legal or otherwise) on vaccine hesitancy. The Urban Institute explored differences in California between adults in families with people born outside the USA or living with a foreign-born family member and adults who are not living in immigrant families [53]. They discovered that adults in immigrant families (75%) showed intention to be vaccinated more than non-immigrant adults (68%). However, they were more likely to report that their regular source of healthcare was a clinic or health center as opposed to a doctor’s office. This may be due to an increased uninsured rate in adults in migrant families. Immigrant families described difficulties with accessing information about COVID-19 and the vaccines, language barriers, and conflicts between work and clinic hours.

Concerns among undocumented immigrants usually centered on the lack of access to information and fears over their precarious status in the USA. For instance, many undocumented residents feared being asked for ID and insurance information that they may not have. Also, they believed the information would be used to contact immigration authorities such as the U.S. Immigration and Customs Enforcement (ICE) and U.S. Customs and Border Protection who they fear may be waiting for them at the clinic [54–61]. In addition, the Trump Administration’s redefinition of “public charge,” which certified that use of public assistance services may be cause for rejection of residency, concerned many non-citizens as they are worried that the free publicly available vaccine would be included in this definition of a public charge [55, 57, 61–63]. Immigrants were also afraid of the cost of the vaccine despite the vaccine being free and the potential residual costs involved if the side effects affected their ability to work [55, 58, 63, 64]. Theories concerning DNA alteration of forced sterilization were commonly discussed among Hispanic immigrant communities as well [54, 59, 65]; however, we did not find evidence that this was salient discussion topic among other immigrant communities.

While a focus on Hispanic communities demands attention other migrant communities also need to be considered. For example, African immigrants have fears that the vaccines may harm them, as advised from friends and family living in their country of origins—having African and African-Caribbean health workers provide information in various African languages have proven useful in educating African migrants [66]. Indeed, addressing these language needs and collaboration with community organizations will be important in reducing vaccine hesitancy [67].

### COVID-19 Vaccine Access

Attributing racial and ethnic disparities in vaccine uptake to mistrust in government and the medical establishment is incomplete. In earlier sections of this article, the proportion of Black and Hispanic people interested in becoming vaccinated increased over time. Yet, the proportion of persons vaccinated has remained lower among Black and Hispanic Americans than White Americans.

There are many elements relating to access such as convenience in location in place and hour. The University of Pittsburg School of Pharmacy geocoded access to vaccine administration facilities nationally by county and this provides information to show inequitable access to sites where vaccines can be administered [68]. They identified 68,128 potential COVID-19 vaccine administration facilities, including 51,207 community pharmacies, 12,464 federally qualified health centers, 3191 hospital outpatient departments, and 1266 rural health clinics (with an overall average of 2.8/10,000 residents). They also identified 94 counties (predominantly in 7 states) where Black residents had a significantly higher risk than White residents of having a driving distance greater than 10 miles to the closest facility and another 69 of greater than 1 mi.

Even when vaccine distribution centers are more evenly distributed, researchers found that racialized minority communities are still experiencing inequitable access [69]. They found there are a variety of reasons why this may be, including individuals from outside of these neighborhoods accessing the vaccination programs [69]. Online registration systems were partly to blame because there was often a racial divide in who had access to reliable internet. For example, in Washington, D.C., wealthier White people reserved virtual appointments in neighborhoods not their own, which limited appointments available to Black residents. The city attempted to address this by implementing a new system based on ZIP codes at highest risk for COVID-19 infection and death. A more complete accounting of vaccine access is the topic of a separate paper from our group.

## Conclusion

Immediately, prior and early during vaccine roll-out, issues of confidence in the COVID-19 vaccines were a concern that crossed racial and ethnic groups. Some of the reason expressed included a “wait and see” for FDA approval or to see how others responded [21, 70]. Other reasons behind vaccine hesitancy were diverse, and varied across demographic groups (Table 2). Combined, these data suggest the importance of communication channel that provide clear messaging about the development and planning for the vaccine, precision planning for logistics of roll-out, and a structure that convenes stakeholders to plan and activate vaccine roll-out through an equity lens.

This review is not without limitations. Most of the studies to date present and focus on univariate analyses by demographic characteristics such as race, ethnicity, age, sex/gender, geography, and political affiliation. Future studies will need to become more nuanced including understanding the impact of intersectionalities and their associated structural marginalization on COVID-19 vaccine acceptance and exploring systemic barriers to vaccine uptake such as access [69].

Since the experience of the first few months of vaccine roll-out, there has been progress in vaccine uptake among Black and Hispanic persons; yet, disparities persist [71]. As the roll-out of vaccine progresses, we can gain a fuller perspective of what interventions and activities influenced eventual uptake. Yet, this rapid review [72] which focused on the first months provides insights on salient issues early in the COVID-19 vaccine roll-out that likely shaped attitudes, intent, and behaviors that contributed to delay in uptake. Our next report reviews types, experiences, and the limited evaluation of interventions started in the early months of vaccine roll out that addressed hesitancy and access.
